# Hernia

**Published:** 1828-05

**Authors:** 


### HERNIA.

### *Strangulated Hernia in an Infant twenty days old: Operation.* Treated by M. DUPUYTREN, at the HOTEL DIEU.

ON the 14th of March, a male child, twenty days old, was admitted, presenting all the symptoms of a strangulated hernia: a shining and painful tumor in the bend of the right groin, descending to the scrotum, nausea, hiccough, stercoraceous vomiting, but not a complete suppression of stools. Leeches were applied; the infant was put into the warm bath, without success. The mother declared that this condition had lasted for three days. M. SANSON, who caused the child to be brought to the hospital, had no doubt of the existence of a hernia. M. DUPUYTREN examined the case with great care: the taxis caused a portion of the tumor to re-enter, which reappeared the moment the compression of the fingers was removed. Uncertain as to the line of conduct that ought to be pursued, M. Dupuytren caused the infant to be taken to the amphitheatre, and proceeded to a fresh examination of the parts, preceding it by some general considerations, of which the following is a summary:

Inguinal hernias, according to the Professor, are not very uncommon at this age, and even in female children the proportion of these hernias is greater than of crural, which he thinks may be attributed to the want of development in the pelvis; but what may be considered as very rare at so tender an age, is a strangulation so marked as to call for an operation. The present is the only instance of the sort which M. Dupuytren has met with in so young a subject. The testicle appeared to have descended into the scrotum; it is situated at the lower and posterior part of the tumor, which at its lowest part appears to contain a fluid, which extends along the chord as far as the superior part of the inguinal canal, which it entirely fills.

Some cases, in which, from the symptoms, the existence of a strangulated hernia has been suspected, and in which the operation has proved the mistake of the surgeon, must be taken into consideration: three or four times a swelling of the chord has imposed upon the operator. Lately, indeed, the Professor was called upon by M. Husson to visit a female in one of the wards of this hospital, who suffered from the symptoms of strangulated hernia, and who had in her right groin a voluminous tumor, in which M. Husson believed that he discovered a fluctuation, and which he thought to be an abscess. M. Dupuytren, being of the same opinion, opened the tumor: an enormous quantity of pus escaped, and the apparent symptoms of strangulation disappeared.

In the case at present under consideration, the strangulation, if it exists, is made by the neck of the sac, and the intestine must have escaped at the moment that the testicle passed through the ring, and carried with it a portion of the peritoneum forming the tunica vaginalis. After a moment's hesitation, M. Dupuytren decided upon operating, this appearing to afford the only chance of cure, and not being likely, in the event of a mistake, to aggravate the infant's state. But before he did any thing to the upper part of the tumor, he proposed to make an incision in the lower portion, opposite to the spot where he felt the fluctuation: the tumor once opened, if the intestine was found, it would not be difficult to prolong the incision, and disentangle it.

This incision was made, and gave issue to a quantity of serum: the testicle appeared bare, known by its white colour; a knot of intestine appeared above, its brownish colour contrasted with the white of the testicle, and left no doubt as to its nature. The incision was then prolonged upwards, and the whole tumor laid open: the intestine was kept down by the fingers, and the division of the stricture made with a blunt-pointed bistoury directly upwards. But here an unexpected difficulty presented itself: the infant continued to cry incessantly, and thus to contract its abdominal muscles, and it became impossible to reduce the intestine, which even escaped in greater quantity. The difficulties in reducing the intestine, the necessity of renewing the attempt several times, the small extent of the parts, and the muscular contractions of the child, rendered this operation long and painful. Before and during the operation, the child had several abundant stools.

After the operation, the fœcal evacuations became perfectly re-established; there were no longer hiccough, tension, vomiting, nor pains in the abdomen; but, whether the first dressing was made without due care, or whether the necessity of retaining the parts within the abdomen induced the operator to tighten the bandage too much at the upper part, to the neglect of the lower, we know not, but, when the dressing was removed, the testicle was found outside of the wound: the house surgeon could not replace it. It had contracted adhesions, which M. Breschet would have destroyed, but an erysipelas had developed itself round the wound, upon the thighs, legs, and loins. M. Breschet inquired whether straps of adhesive plaster had been applied, to which the erysipelas might well have been attributed, but they had not; and therefore it became a question to what it was owing. It appears probable that inflammation was produced by the compression of the testicle:

its appearance out of the wound, its contact with the external air, and the dressings, might have contributed to it: had that been the case, perhaps it would have been right to have destroyed these adhesions at once, and to have reduced it directly, in spite of the inflammation to which its protrusion had given birth, and which tended to support it. However this might be, leeches, emollients, and two blisters to the legs, diminished the erysipelas, and induce a hope of the fortunate termination of the case.

---

*Case of Scrotal Hernia, attended with some peculiar circumstances.*

William Cooper, æt. forty-seven, was brought to the Middlesex Hospital, at five o'clock in the morning of the 10th of February, by a surgeon who had previously attended him. He had a scrotal hernia on the right side, which, on his being placed in bed, was reduced with the greatest ease.

It was then stated that the patient had been subject to hernia for twenty years; that it had come down five years ago, and there had been some difficulty in reducing it. After that time he wore a truss, but continued the use of it for a short time only. On Thursday last (the 7th,) in the evening, his rupture again came down; he felt sick, and took a black draught, which he vomited. Next day he sent for a chemist, who made several attempts to reduce the hernia: upon his not succeeding, he confessed that he had not much experience in these cases, and left the patient. About two o'clock in the afternoon, he was seen by the surgeon who accompanied him to the hospital. This gentleman succeeded in reducing the hernia, so that the tumor disappeared, and he could even push the point of his finger into the external abdominal ring. The patient expressed himself greatly relieved after this operation. He obtained no evacuation from his bowels, however, from the time that the hernia came down until he was conveyed to the hospital. This surgeon gave him, in separate doses, to the amount of fifty grains of the compound extract of Colocynth, and sixteen grains of Calomel, and several injections by the rectum. He also bled him, soon after the operation, to twenty ounces.

When brought to the hospital, his condition showed that he was in a state of great danger. He had constant vomiting; his abdomen was swelled and tympanitic, and exquisitely tender to the touch, particularly at the lower part, on the right side; he had a small, quick, almost a fluttering pulse; his features were sunk and pallid. The house surgeon ordered him a dose of Castor-oil with Laudanum and a clyster. When visited at ten o'clock, he had had three motions, and expressed himself as being a great deal better, and quite easy. His pulse was fuller.

During the greater part of the day, he continued to feel easier than he had hitherto been. But, about six o'clock in the evening, it was found that his extremities were cold and damp; he was restless, complaining of pain in his abdomen. He died that night. It is to be remarked that the hernia came down repeatedly during the day, and was each time reduced with great facility.

*Dissection.*—The hernia was in the scrotum: it had come down shortly before death. Upon cutting through the abdominal muscles, the intestines rolled out from the incision, being distended with flatus. All the small intestines were highly inflamed, distended to the utmost, and in some parts loaded with dark fluid contents. The portion of gut which was included in the hernial sac was a knuckle of the intestinal ileum, very near its termination in the cœcum. Above the stricture, the intestine was of a deep red colour, marked with several patches of an inky blackness, and it was loaded with dark offensive fluid. On turning these coils aside, the lower portion of the ileum, leading from the hernia towards the cœcum, was seen small and contracted, its folds being in a mass together. These were of a pale green colour, and their surfaces were marked here and there with dark mortified spots. In the colon there was nothing to remark, except that it was contracted, and that it had not partaken of the inflammation. There was a large duplicature of the transverse arch, with a thickened mass of omentum attached to it, which appeared, from its form and the old adhesions that united it, to be the portion which had been reduced five years ago, when the rupture had come down.

